# Integrating information in the brain’s EM field: the cemi field theory of consciousness

**DOI:** 10.1093/nc/niaa016

**Published:** 2020-09-22

**Authors:** Johnjoe McFadden

**Affiliations:** Faculty of Health and Medical Sciences, University of Surrey, Guildford, Surrey GU2 5XH, UK

**Keywords:** cemi field theory, computing, integrated information, consciousness, electromagnetic field, qualia

## Abstract

A key aspect of consciousness is that it represents bound or integrated information, prompting an increasing conviction that the physical substrate of consciousness must be capable of encoding integrated information in the brain. However, as Ralph Landauer insisted, ‘information is physical’ so integrated information must be physically integrated. I argue here that nearly all examples of so-called ‘integrated information’, including neuronal information processing and conventional computing, are only temporally integrated in the sense that outputs are correlated with multiple inputs: the information integration is implemented in time, rather than space, and thereby cannot correspond to physically integrated information. I point out that only energy fields are capable of integrating information in space. I describe the conscious electromagnetic information (cemi) field theory which has proposed that consciousness is physically integrated, and causally active, information encoded in the brain’s global electromagnetic (EM) field. I here extend the theory to argue that consciousness implements algorithms in space, rather than time, within the brain’s EM field. I describe how the cemi field theory accounts for most observed features of consciousness and describe recent experimental support for the theory. I also describe several untested predictions of the theory and discuss its implications for the design of artificial consciousness. The cemi field theory proposes a scientific dualism that is rooted in the difference between matter and energy, rather than matter and spirit.

## Introduction

‘Love is a smoke made with the fume of sighs’

William Shakespeare, Romeo and Juliet

‘What’s the best way to fix a bicycle that has a rope caught in its spokes?’

Gary Marcus ‘Deep Learning: A Critical Appraisal’ (Marcus 2018)

The ‘binding problem’ is that of understanding ‘our capacity to integrate information across time, space, attributes, and ideas’ (Treisman 1999) within a conscious mind. The problem is often posed in terms of understanding how the disparate components of a visual scene—colours, textures, lines, motion, etc.—that are processed in distinct regions of the brain are yet brought together to form a unified conscious percept. However, binding is a general feature of consciousness in all its modes. The first text quotation above contains four discordant nouns, one denoting an emotion, the second a dark vapour, the third a noxious smell and the fourth, an utterance. Yet, Shakespeare’s genius bound each word into a single line of poetry that effortlessly evokes, in the conscious mind of the reader or listener, a singular, integrated, yet complex insight into the most tender of human emotions. In the second quotation, artificial intelligence (AI) researcher and pioneer of Deep Learning, Gary Marcus, laments the fact that AI currently lacks this ability, as illustrated by the intractability of problems, such as untangling a rope from the wheel of a bicycle that is, nevertheless, grasped and solved by any infant on her first exposure to the task.

Our subjective experience is that this kind of problem, which involves planning and executing several sequential steps, is nonetheless instantly grasped and solved in its entirety, as integrated information. This intuition is borne out by many studies that demonstrate that the binding provided by consciousness is indeed required to solve general intelligence problems, particularly sequential tasks that require working memory, such as memory trace conditioning (Carter *et al.* 2006), multi-step calculations (Dehaene and Cohen 2007), goal-directed behaviour and strategic planning (Dehaene and Naccache 2001), learning over time (Fuster 1991), language (but not word) comprehension (Hagoort and Indefrey 2014), social intelligence and interactions (Dunbar *et al.* 2010; Lieberman 2012) and creativity (Kaufman *et al.* 2010). As has been pointed out by several researchers (Tononi and Edelman 1998; Treisman 1999; Edelman and Tononi 2008), conscious binding requires the integration of complex information in the brain. The problem is to understand how the brain achieves this integration.

## Results

### What do we mean by physically integrated information?

‘Philosophy is a battle against the bewitchment of our intelligence by means of our language’. Wittgenstein (2009, p. 109).

What do we mean by ‘integrated information’? To answer this question, we must first agree on a definition of information. I will here use that described by Claude Shannon and known as ‘Shannon information’ (Shannon 1948); which is essentially a measure of correlation between the degrees of freedom of a sender and receiver of a message, measured in bits. Neuronal firing rates thereby encode information about the outside world because some of its degrees of freedom are correlated with degrees of freedom of the outside world. I note that, in some theories of consciousness, causation is required in addition to correlation (Landauer 1991; Tononi *et al.* 1998).

Next, we must agree on how to distinguish conscious from non-conscious mental activity. I will follow the approach pioneered by Dehaene and colleagues who insisted that ‘subjective reports are the key phenomena that a cognitive neuroscience of consciousness purport to study’ (Dehaene and Naccache 2001). So, bringing these two definitions together, then conscious neuronal information (the sender) is that information encoded in the brain that correlates with the information encoded in the subjective reports (the receiver) of a conscious observer.

However, a great deal of information, as degrees of freedom in the brain, may be correlated with subjectively reported information, including the motion of ions through neuronal membrane, the motion of neurotransmitters within the synaptic cleft, the opening and closing of ion channels, blood flow or electromagnetic (EM) field perturbations generated by the motion of electrically charged particles. Each of these neuronal microstates *knows*, in the Shannon sense of its state being correlated with, some aspect(s) of the visual scene or subjective reports of that scene. Which is a likely physical substrate for the integrated information that must be encoded by conscious minds?

Before answering this question, it is first necessary to define what we mean by integrated information. This might appear to be an easy task as the term is widely used, so much so that there is evidenced by the United Nations Expert Group on the Integration of Statistical and Geospatial Information (http://ggim.un.org/UNGGIM-expert-group/), the International Society of Information Fusion (http://isif.org/), Information Integration Theory (Anderson 2014), data integration systems (Genesereth *et al.* 1997), numerous statistical and data mining methods that seek to integrate information from multiple sources (Maimon and Rokach 2005), as well as the integrated circuits of computers. However, as the physicist, Rolf Landauer, famously insisted, ‘information is physical’ (Landauer 1991) so integrated information, if it exists at all, must be encoded by a physically integrated substrate. Moreover, if it is to have an output then it needs to be causally competent (Pawłowski *et al.* 2009): the integrated information must, as an integrated unit, change something physical. Yet, none of the above examples of integrated information are physically integrated. Their information is causally integrated in time, rather than physically integrated in space, as I will now illustrate with a familiar problem from philosophy.

Ryle (2009) insisted that it is a category error to suppose that structures, such as the University of Oxford, have material existence. To make his point, he imagined a visitor to Oxford who visits the library and colleges but then asks ‘But where is the University?’ The visitor’s error is to assume that the university is a member of the category of material objects, rather than an institution which exists causally only in the minds of the students, staff and visitors to the university. Ryle calls this kind of mistake a ‘category error’. An analogous argument can be made for integrated information. University institutions, such as registry, finance, the libraries, exam boards, colleges or executives, integrate and process vast amounts of varied information ranging from student entry criteria, applicant qualifications, book catalogues, exam performance, timetables or salaries. However, this integration, like the institution itself, is causal, rather than physical, in the sense that downstream effects, such as the posting of offers of university places, depend on a multiplicity of upstream informational causes, such as the arrival of application forms and their scrutiny by academics and administrators. The integration is via a causal chain of operations in time, rather than physical integration in space.

**Figure 1:**
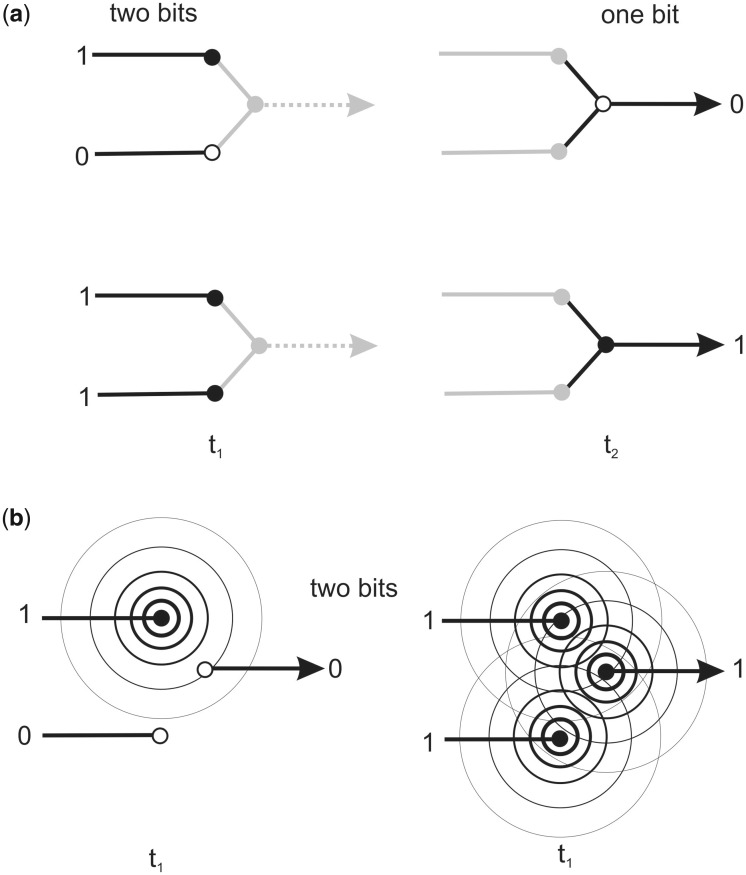
(a) Illustration of how the single bit output of an AND logic gate integrates the two bits of information encoded in its two inputs to output a single bit that represents an integration of the gate's inputs. In reality, the integration is causal in the sense that the state of the output bit at time t2 is dependent on both input bits at time t1. (b) Illustration of how dynamic EM field information can integrate information and function as a logic gate. An AND gate is shown with two inputs and a single output, each encoded either a zero or one all at time *t*_1_. The inputs are dipoles that act as electromagnetic field (EMF) transmitters that oscillate between two states either firing (oscillating corresponding to input = 1) or non-firing (not oscillating, input = 0) states. The output is then an EMF receiver that implements the AND rule to output a signal.

This form of temporal integrated information is also a universal feature of computation, such as those performed by Boolean logic gates and instantiated in Turing machines, such as modern computers. For example, the single bit output of an AND logic gate integrates the two bits of information encoded in its two inputs (Fig. 1a) to output a single bit that represents an integration of the gate’s inputs. In reality, the integration is causal in the sense that the state of the output bit at time *t*_2_ is dependent on both input bits at time *t*_1_. Of course, the single output bit cannot encode both input bits: it is not physically integrated information. Note also that, once a signal has been transmitted from input to output via, for example, a current or voltage change, then the inputs are free to accept new data. So, by the time the signal has reached the output (*t*_2_), its state may no longer be correlated with the inputs. There is therefore no physical state that corresponds to the integration of information performed by the gate. This does not, of course, prevent the gate from participating in any complex algorithmic operation that can be considered to *integrate* information in the sense of the conventional temporal use of the term. Consider, for example, a computer connected to a camera and running an image recognition program whose job is to identify photographs of the actress Jennifer Anniston amongst hundreds of electronic images presented to the camera. By the time the complex series of computations that analyse and integrate features such as hair colour, eye colour, shape of nose, chin, complexion, orientation of the image, etc., have reached a gate that finally commits the program to deliver an audio output of ‘this is Jennifer Aniston’, the input logic gates may have gone on to the next photograph. There can therefore be no physical state that corresponds to the integrated information at any single point in time. The integration exists as a correlation in time, not as a physical integration in space.

### Do neurons integrate information?

It is important to stress that no EM field theory of consciousness denies that much or most brain information processing proceeds via conventional neuron/synapse transmission. However, the same argument described above for integrated circuits, applies to the processing of complex information along complex neuronal pathways. They, like logic gates, input sensory information, such as photographs, and process that information along chains of neuronal networks until they reach a group of neurons, or even a single neuron that fires to generate a verbal output of ‘this is Jennifer Aniston’. Nevertheless, just as for logic gates, once a neuron has done its job of processing its many inputs to generate the single output of a firing rate, it is free to accept new data. Downstream neurons involved in the triggering motor output need not be correlated with the simultaneous state of input neurons or neurons involved in the processing of that sensory information by the time the output signal is generated. A hypothetical neuron that prompts the final output of a verbal report is also no more physically linked to its sensory inputs such, as in the retina or ear, than it is to a subject’s digestive system or skull. And even neurons that respond to highly processed and integrated information, such as the famous Jennifer Aniston neuron (Quiroga *et al.* 2005), only encode a single firing rate that cannot represent anything more than a tiny fraction of the information present in conscious percept. Its firing rate is, of course, all that any single neuron can *know*. Neurons integrate information but, as for logic gates, the integration is temporal, not physical, as the information to be integrated is separated in both space and time. Also, the so-called integrated information is far more complex than a single neuron’s coding capacity. This kind of temporal integration cannot correspond to ‘our capacity to integrate information across time, space, attributes and ideas’ (Treisman 1999).

Of course, a huge number of firing neurons may fire in response to Jennifer Aniston’s face or some other potentially integrated conscious percept. It may be that each of these firing neurons encodes different aspects of the conscious percept—perhaps the particular colour of the actress’ eyes, the shape of her nose or the texture of her hair. One could imagine a network of such neurons that, together, encode and ‘integrate’ the conscious percept. But how do the firing neurons integrate their information? From the perspective of functionality, they need not even be physically connected so long as they, collectively, deliver their signals to a motor neuron(s) that execute(s) the verbal report ‘that is Jennifer Anniston’s face’. In reality, they need be no more physically connected to each other than are the different colleges of the University of Oxford. All that is required is a causal chain.

Nevertheless, nerves are, of course, physical connected, but, in the example, those encoding Jennifer Anniston’s face are no more physically connected to each other than they are to any other neurons or even cells of the body, particularly as matter-based signals, such as hormones, neurotransmitters or action potentials, do not attenuate with distance. If physical connectedness was sufficient for consciousness, then we would be aware of all of the information encoded in our entire body at all times. Neural networks, on their own, cannot be responsible for physically integrating conscious information because, like integrated circuits, they integrate information only temporally, not physically.

### Integrating information in space, rather than in time

There are, however, physical systems that encode information integrated over space in a single moment of time. We know this form of information as force fields. The most obvious is the gravitational field that, at any point on the Earth’s surface, provides a force that effectively integrates the magnitude and distribution of local masses such as those of the Earth, Moon and Sun. Similarly, the EM field at any point in space represents an integration of information concerning the type, distribution and motion of local charges. In contrast to the temporal integration described above, force fields physically integrate complex information that may be simultaneously downloaded from any point in the field. This is apparent to anyone who views a TV show that has been transmitted from a single transmitter to their smartphone, alongside a thousand other people who may simultaneously view the same program on their phones in a thousand different locations. Moreover, an EM field can, like an integrated circuit, compute.

Consider for example, the arrangement of iron filings sprinkled over a magnet. A conventional computer could calculate their configuration by inputting the initial random configurations of the filings into an algorithm that implements either Maxwell’s equations or the equations of quantum electrodynamics to output their final equilibrium configuration. Yet the EM field at each point in space, generated by the electron spin of iron atoms within the magnet, instantly *computes* this solution. In this sense, the field represents an algorithm in space, rather than the algorithms in time that are implemented by Turing machines. And, most importantly, the information involved in the computation is simultaneously available in the space of the magnet and its surroundings. It is spatially, rather than temporarily, integrated information. The EM field’s information is complex information that is physically *bound*.

**Figure 2.**
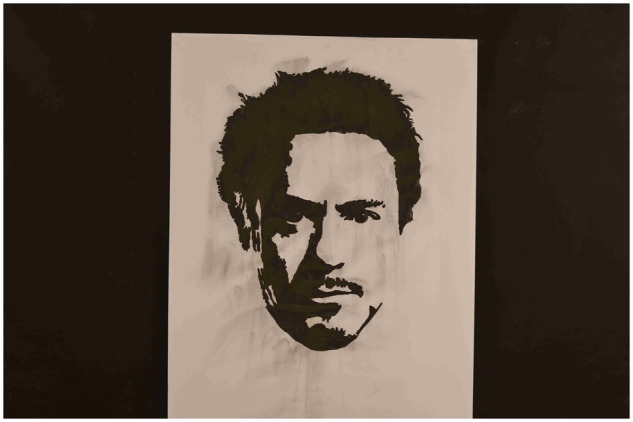
Magnetic portrait of the actor Robert Downey Jr by the artist, Andrzej Lenard.

Magnets can also encode visual information. The artist, Andrzej Lenard, paints magnetic pictures, such as the portrait of Robert Downey Jr (Fig. 2 from https://www.youtube.com/watch? v=PHzz81yapcc). Note that the magnetic field-encoded information would be present whether the iron filings were there or not. The coding of the image would exist in space as invisible integrated information. This kind of coding is, I argue, much closer to the physical reality of our thoughts, than a firing neuron. However, it is static, rather than dynamic, and, unlike the signal from a WIFI router, the visual information is locally discrete rather than distributed throughout the space of the image.

**Figure 3.**
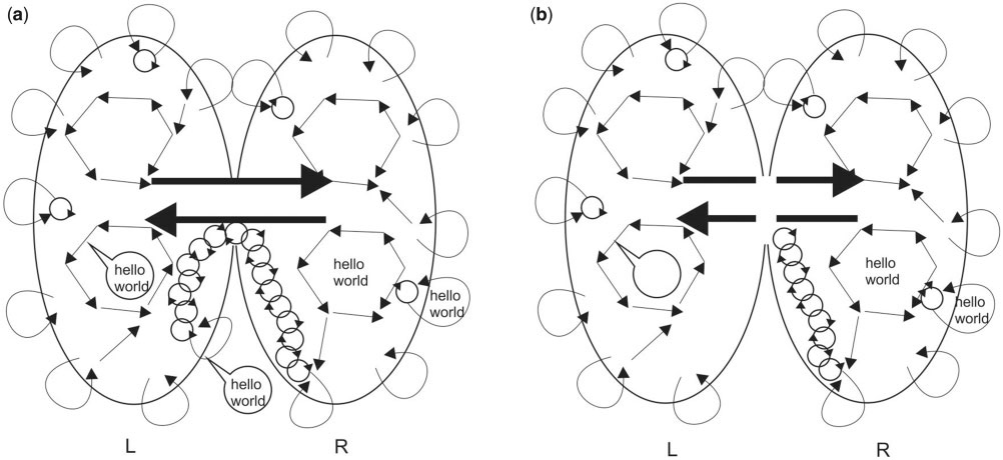
(a) Schematic of the two hemispheres of the brain connected by the corpus collosum bundle of nerve fibres (double-headed arrow). Each hemisphere has its own neuronal/synaptic connections represented by straight lines and closed circle arrows representing recurrent neuronal pathways whose synchronously firing networks generate conscious EM field perturbations represented by curved arrows. By recruiting adjacent neurons into oscillations, waves of synchronously firing neuronal networks—and thereby conscious thoughts—can travel large distances through the brain, for example, from the right hemisphere, via the corpus callosum, to speech centres (speech bubble) in the left hemisphere. This allows conscious thoughts (‘hello world’) originating in the right hemisphere to communicate to the outside world through speech. In contrast, being subject to an inverse cube law, EM field perturbations are highly local so are incapable of transmitting across long distances unless reinforced by relays of synchronized neurons forming recurrent networks. (b) Severing the corpus callosum prevents transmission of conscious thoughts from the right to the left hemisphere since they can no longer be transmitted, via recurrent networks of neuronal oscillations, through the corpus callosum.

Figure 1b illustrates how dynamic EM field information can integrate information and function as a logic gate. The figure illustrates an EM field AND gate with two inputs and a single output, each encoding either a zero or one. The inputs are dipoles that act as EMF transmitters that oscillate between two states. For ease of presentation, Fig. 1b illustrates the different inputs represented as firing (oscillating corresponding to input = 1) or non-firing (not oscillating, input = 0) states. The output is then an EMF receiver that implements the AND rule to output a signal. Note that the receiving node can be located anywhere in the space of the field. So, and most importantly, the entire AND computation is distributed throughout the space of the overlapping field of the inputs. The computation and integration of input information is implemented in space, rather than time. It is physically integrated information processing.

Combinations of EMF transmitters and receivers that implement different logical functions could, in principle, implement any complex algorithm, for example, an algorithm that recognizes images of Jennifer Aniston. In contrast to the above static magnetic image of Robert Downey Jr, this EM field algorithm would be distributed throughout the space of the field; such that, like a WIFI signal, it could be downloaded from any point in the space of the field. In this sense, the field possesses features in common with holograms that similarly store distributed information. But in the case of the cemi field, the information exists as an algorithm in space, rather than time. It is physically integrated information. Field-implemented algorithms such as these, but in the brain, are, I argue, the physical substrate of conscious thoughts.

It is, however, important to recognize that, although EM fields are maximally connected—the universe has a single EM field EM – waves travel at the speed of light across huge distances. However, their strength is subject to an inverse square (electric component) or cube (magnetic component) law so that the EM field perturbation of a single neuron rapidly falls off with distance. In my earlier paper, I estimated that the EM field electrical perturbation from the firing of a single neuron extends into a volume of only about 80 µm encompassing a maximum of about 200 neurons (McFadden 2002a). So, in contrast to matter-based signals that do not attenuate with distance, signals passing through the cemi field will tend to act only locally, unless boosted by chains of synchronization (see below).

Note however, and very importantly, that, in contrast to temporally integrated information, an algorithm in space can function only when its computational nodes fire synchronously so that their inputs are simultaneously available to all the components of the network. Therefore, a key prediction of the proposal that consciousness is distributed EM field-based algorithms is that conscious information will be correlated with synchronously firing neurons.

### EMF transmitters and receivers in the brain

It has been known since the 19th century that the brain generates its own EM field, which can be detected by electrodes inserted to the brain. Its source is electrical dipoles within the neuronal membranes caused by the motion of ions in and out of those membranes during action potentials and synaptic potentials. The periodic discharge of neurons—firing or action potentials—generates EMF waves that propagate out of the neuron and into the surrounding inter-neuronal spaces where they overlap and combine to generate the brain’s global EM field that is routinely measured by brain scanning techniques such as electroencephalography (EEG) and magnetoencephalography (MEG). The human brain therefore possesses around 100 billion EMF transmitters.

The human brain also possesses at least 100 billion EMF receivers as each neuron in bounded by a membrane embedded with thousands of voltage-gated ion channels whose firing is triggered by EM field fluctuation across the membrane. Although these channels are generally assumed to respond only to large fluctuations of tens of millivolts across the membrane, much larger than the global EM field strength, EM field potential changes of less than 1 mV across the neuronal membrane are nevertheless capable of modulating neuronal firing (Schmitt *et al.* 1976). Moreover, for neurons poised close to the critical firing potential, the opening of just a single ion channel may be sufficient to trigger firing (Arhem and Johansson 1996). This degree of sensitivity suggests that very tiny changes in membrane potential, of similar strength to spontaneous fluctuations in the brain’s endogenous EM field, may influence the firing of neurons that are already close to firing.

### The cemi field theory of consciousness

The conscious electromagnetic information (cemi) field theory claims that the brain’s EM field is the physical substrate of consciousness. It was first outlined in a book published in 2000 in which I proposed that the brain’s ‘EM field … integrate[s] information from all of the calculations … performed by … [its] logic gates (McFadden 2000). The theory was further developed in two papers published in 2002 (McFadden 2002a,b). Similar theories were proposed around the same time by neurobiologist Pockett (2000, 2002), the neurophysiologist John (2001, 2002) and the neurophysiologists Fingelkurts and Fingelkurts (2008) and Fingelkurts *et al.* (2001). In 2013, I provided an update on the cemi field theory incorporating more recent experimental evidence (McFadden 2013a) as well as arguing that the theory accounts for the gestalt properties of meaning, in an accompanying paper (McFadden 2013b). In 2014, Adam Barrett similarly argued that the brain’s EM field integrates neuronal information to provide the substrate of consciousness (Barrett 2014).

The idea that the seat of consciousness is simply the brain’s EM field may initially sound outlandish but is no more extraordinary than the claim that the seat of consciousness is the matter of the brain. All it involves is going from the right to the left hand side of Einstein’s famous equation, *E* = *mc*^2^ thereby replacing the notion that consciousness is encoded by matter of the brain, with that of proposing that it is encoded by the energy of the EM fields generated by the motions of its charged matter. (Note that, by illustrating this idea with Einstein’s equation, I am not, of course, proposing any interconversion of matter and energy in the brain.) Matter and energy are equally physical; but, instead of being composed of material, the cemi field theory proposes that our thoughts are composed of the brains EM field energy. This is a kind of dualism, but it is scientific dualism based on the physical difference between matter and energy, rather than a metaphysical distinction between matter and spirit.

Although, as I have argued, shifting from matter to energy of the brain is conceptually trivial, when searching for an appropriate substrate in the brain that can physically integrated complex information, the move draws an immediate payoff, as it effortlessly solves the binding problem. Whereas information encoded in the matter of neurons is, as I have argued, always localized and discrete both in space and time, information in the field, as illustrated in Fig. 1b, is always integrated yet distributed, in the sense that it may be downloaded from any point within the field. Since, in this case, ‘the field’ is the brain’s global EM field, it also provides a feasible physical substrate for the notions of working memory and/or the global workspace that have been proposed in many other theories of conscious (Baars 2005). And, as illustrated in Fig. 1(b) EM fields may also implement algorithms. This capacity, known as ‘field computing’ (MacLennan 1999) sometimes as quantum-like computing (Khrennikov 2011), has several features in common with quantum computing such as ease of implementation of mathematical functions such as Fourier transforms, compared to digital computers. Moreover, as illustrated in Fig. 1b, this form of field computing—algorithms in space rather than in time—could only be implemented by neurons (either EMF transmitters or receivers) that fire synchronously. So, the theory predicts that, if consciousness is implementing field computing, then consciousness should be highly correlated with the synchrony of neural firing rather than firing rates.

Several decades ago, work conducted by Wolf Singer and colleagues demonstrated that neurons in the monkey brain fire synchronously when the animal attends to the stimulus (Kreiter and Singer 1996). Many additional studies confirmed and extended these findings to many different experimental systems. For example, work in David Leopold’s laboratory at Max Planck Institute for Biological Cybernetics, in Tubingen, Germany (Wilke *et al.* 2006) investigated awake monkeys trained to respond to a visual stimulus—the removal of a red dot from a target area—by pulling a lever (to receive their fruit juice reward). The researchers monitored both neuron spiking and changes in local EM field potentials in V1, V2 and V4 regions of the monkey’s visual cortex. They demonstrated that spiking of neurons in cortical areas V1 and V2 was totally uncorrelated with the monkey’s perception of the target; however, low frequency (alpha range, particularly 9–30 Hz) modulation of local field potentials—presumed to be generated by synchronously firing neurons—in these same regions did correlate with perception. It seems that though neuron firing rate in the primary visual cortex does not *see* the stimulus, synchronicity of neuron firing does indeed *see* the target.

Many subsequent studies have also demonstrated that neural synchrony also correlates with conscious perception in humans. For example, neural synchrony patterns were found to correlate with conscious recognition by subjects exposed to optical illusions (Lutz *et al.* 2002). More recent work has demonstrated that conscious auditory perception is correlated with long-range synchrony of gamma oscillations (Steinmann *et al.* 2014). Synchronization between the anterior and posterior cortex has been shown to correlate with consciousness levels of patients who have suffered traumatic brain injury (Leon-Carrion *et al.* 2012).

Of course, there may be several different and often contradictory signals being simultaneously projected into the cemi field by networks or clusters of synchronizing neurons. Even so, what is distinctive about the cemi field in contrast to many other theories of consciousness is that, because EM fields are always unified, there is only ever one EM field in the brain. The dominant information in consciousness will then be the one that is associated with the strongest EM field perturbation capable of modulating neural firing within that singular field. This has been demonstrated in numerous studies, for example this 2005 study (Doesburg *et al.* 2005) demonstrated that increased gamma-band synchrony predicts switching of conscious perceptual objects in classic binocular rivalry. Similar switches in EEG or MEG patterns have been shown to predict conscious percepts in numerous studies (Sterzer *et al.* 2009) opening the possibility of ‘mind-reading’ by decoding brain EM field signals. In nearly all of these studies, the conscious percept corresponds to the dominant EM field signal suggesting that competition between rival percepts is resolved through positive feeding back loops within re-entrant circuits leading to what Dehaene calls a ‘global ignition’ or ‘avalanche’ of the dominant signal (Dehaene 2014). Therefore, in contrast to other theories of consciousness, such as global workspace or Integrated Information Theory (IIT), that use arbitrary or ill-defined thresholds for access to consciousness, the cemi field relies on a measurable physical parameter—the strength of EM field perturbations that are capable of modulating neural firing—to differentiate between conscious and non-conscious brain information.

From a neuronal perspective there appears to be no obvious reason why synchrony should make a difference to neural processing: neurons deliver the same information and perform the same informational processing, whether or not they are firing synchronously. Of course, many theories of consciousness do incorporate neural synchrony by, for example, viewing it as a signature of the re-entrant neural connectivity characteristic of globally distributed neuronal circuits that are proposed to underpin consciousness (Tononi and Edelman 1998; Seth *et al.* 2004), or a consequence of coincidence detection within neurons involved in conscious thoughts. Yet neurons need not fire synchronously to distribute information globally and re-entrant circuits need not, necessarily, fire synchronously. Similarly, there seems to be no obvious reason why conscious neural processing requires coincidence detection any more than non-conscious neural processing as they both perform temporal information integration. So, synchrony, *per se*, is neither a necessary nor sufficient requirement for consciousness in matter-based neuronal models of consciousness. As far as I am aware, it is only in the EM field theories that synchrony plays an obligatory role in consciousness information processing.

Note however that the EM field theories of consciousness are entirely compatible with the observation that highly synchronized brain activity, such as is typical for epileptic seizures, disrupt consciousness. Conscious brain states necessarily encode complex information that correlates with features of the outside world. Widespread neuronal synchrony—such as experienced during an epileptic seizure—is empty of informational content that correlates with the outside world and is thereby only consistent with a non-conscious state.

### ‘Free will’ as the output node of the cemi field

The cemi field theory differs from some other field theories of consciousness (Pockett 2000, 2002) in that it proposes that consciousness—as the brain’s EM field—has outputs as well as inputs. In the theory, the brain’s endogenous EM field influences brain activity in a feedback loop (note that, despite its ‘free’ adjective, the cemi field’s proposed influence is entirely causal (McFadden 2002a)) acting on voltage-gated ion channels in neuronal membranes to trigger neural firing. This assertion is supported by abundant theoretical work and experimental data. Experimental evidence for the brain’s endogenous EM field influencing neural firing was scanty when I first described the theory in 2000 (McFadden 2000) and 2002 (McFadden 2002a,b), but included evidence that transcranial magnetic stimulation (TMS), which generate EM fields in the brain of similar magnitude to the brain’s endogenous EM fields can influence behaviour (Beckers and Hömberg 1992; Amassian *et al.* 1998; Hallett 2000; McFadden 2002a). In 2013 (McFadden 2013a), I summarized more recent experimental evidence obtained from several labs demonstrating that artificially generated external EM fields, of similar strength to those of endogenous brain EM fields, do indeed change firing patterns in whole animals, brain tissue slices and neuronal cells (Fujisawa *et al.* 2004; Frohlich and McCormick 2010; Anastassiou *et al.* 2011). Since then, a wealth of additional experimental evidence has accumulated which clearly demonstrates that the brain’s endogenous EM fields do indeed play a role in communicating between brain neurons (Qiu *et al.* 2015; Anastassiou and Koch 2015; Han *et al.* 2018); prompting some researchers to propose ‘that our visual experience may at least some times be coming through in waves.’ (Mathewson *et al.* 2011).

In summary, there is now, at the very least, abundant evidence that, as well as standard synaptic transmission, brain neurons also communicate through endogenous EM fields. It is a small step from this realization to the cemi field theory, which proposes that the action of the brain’s (conscious) endogenous EM field on neural firing rates is experienced as conscious thoughts that influence our actions. Curiously, the kind of influence proposed for the brain’s EM field in the cemi field theory is very similar to the role proposed for consciousness by William James more than a century ago. James proposed that the cortex is delicately balanced with a ‘hair-trigger’ such that the slightest jar or accident could set it firing erratically, yet ‘if consciousness can load the dice, can exert a constant pressure in the right direction, can feel what nerve processes are leading to the goal, can reinforce and strengthen these and at the same time inhibit those that threaten to lead astray, why, consciousness will be of invaluable service.’ (James 1988, p. 26).

I now discuss how the cemi field theory solves most of the puzzling features of consciousness.

### The difference between conscious and non-conscious brain states

One of the most profoundly puzzling features of the brain is that it operates simultaneously in both conscious and non-conscious streams, at least in man. Most theories of consciousness attempt to account for this difference in terms of some arbitrary critical threshold in the degree of complexity (Seth *et al.* 2006), integration (Tononi and Edelman 1998; Tononi *et al.* 1998; Srinivasan *et al.* 1999), selection (Edelman and Tononi 2008), long distance integration (Dehaene *et al.* 2014) or access to some kind of hypothetical global workspace (Baars 1988; Dehaene *et al.* 1998), that is necessary for conscious awareness. Yet these theories have difficulty accounting for why some highly complex and integrated neuronal activities, such as those involved in decoding grammatical rules within a sentence, are performed without awareness; whereas others that should be much simpler, such as those involved in long multiplication, can only be performed consciously.

In contrast to the threshold models above, once it is accepted that EM fields influence neural firing patterns (as evidenced in TMS and external EM field studies, described above) then the evolutionary emergence of both conscious and non-conscious mental streams becomes inevitable. This follows because the impact of the brain’s endogenous EM field on neuronal computations is likely to be both positive and negative. Positive influences could result from phase-locking of multiple downstream EM field-sensitive neurons to the same stimulus; or rapid distribution of EM-field-encoded information to many regions of the brain (as in the global workspace model). Also, as argued above, ‘field computing’ may provide computation capabilities that are hard to emulate in the neuronal brain. Negative influences of EM fields would include all the varieties of undesirable ‘feedback’ familiar to both sound and electrical engineers. Having both positive and negative influences on brain function, then the brain’s EM field would have become visible to natural selection. Mutations in genes that enhanced sensitivity, perhaps by increasing synchronous firing, in neuronal circuits in which EM fields enhance fitness would have been positively selected; as would genes that decrease EM field sensitivity in neuronal circuits in which EM fields decreased fitness. The brain would then have inevitably evolved into an EM field-sensitive and conscious stream associated with synchronous neural firing; together with and an EM field-insensitive but non-conscious stream associated with asynchronously firing neurons. This is of course precisely what we find in the human mind. Indeed, once it is accepted that EM fields influence fitness both positively and negatively through their impact on neural firing rates—as seems evident from the evidence outlined above—the theory of natural selection predicts that brains will evolve in precisely this way.

The cemi field theory also naturally accounts for the fact that the non-conscious mind appears to operate as a parallel processor that can perform several tasks simultaneously, such as whistling a tune whilst riding a bicycle; whereas the conscious mind appears to operate as a serial computer incapable of, for example, reading whilst simultaneously engaging in a conversation. That the non-conscious mind can operate in parallel is not problematic. With 100 billion neurons at its disposal, it is easy to see how the brain can partition operations amongst them. The puzzle is to understand why conscious tasks always interfere with one another. As far as I am aware, this is not accounted for by any matter-based neuronal theory of consciousness but is easily accounted for in the cemi field theory as the brain’s conscious EM field, as already pointed out, is always singular. Just as tossing two stones into the same still pond will generate waves that interfere with each other; so two thoughts emerging within a brain’s global EM field will always interfere with one another. So, within the cemi field theory, the brain naturally divides into a non-conscious neuronal parallel processor capable of implementing lots of independent tasks without interference; and a conscious EM field-based serial processor that can only do one thing at a time.

I should also point out that, in contrast to other theories of consciousness, such as IIT, although information integration is central to the cemi field theory, the theory does not predict that, as understood in its usual temporal or computational sense, information integration is either exclusive to, or maximal in, consciousness. In fact, it is perfectly possible that the simplest thoughts may sometimes dominate consciousness, consistent with the finding that meditative states are often associated with slower and more rhythmic patterns in EEG (Banquet 1973) and MEG (Dor-Ziderman *et al.* 2013). Conversely, the theory is also consistent with the finding that tasks that require a considerable degree of information integration, such as recognizing words, or whether one number is greater or less than another, may be performed without awareness; whereas more complex operations, such as multiplication or natural language understanding appear to require consciousness (Dehaene *et al.* 2006). The lack of correlation between complexity of information integration and conscious thought is also apparent in the commonplace observation that tasks that must surely require a massive degree of information integration, such as the locomotory actions needed to run across a rugged terrain, may be performed without awareness but simple sensory inputs, such as stubbing your toe, will over-ride your conscious thoughts. The cemi field theory proposes that the non-conscious neural processing involves temporal (computational) integration whereas operations, such as natural language comprehension, require the simultaneous spatial integration provided by the cemi field. The theory is also consistent with the finding that non-conscious neural processing may be more robust to disruption by external EM fields than conscious processing as is evidenced by, for example, the finding that TMS to V1 induces blindsight (Dehaene *et al.* 2006).

### Signatures of consciousness

Dehaene (2014) has recently described four key signatures of consciousness: (i) a sudden ignition of parietal and prefrontal circuits; (ii) a slow P3 wave in EEG; (iii) a late and sudden burst of high-frequency oscillations; and (iv) exchange of bidirectional and synchronized messages over long distances in the cortex.

It is notable that the only feature common to each of these signatures—aspects of what Dehaene calls a ‘global ignition’ or ‘avalanche’—is large endogenous EM field perturbations in the brain, entirely consistent with the cemi field theory. It is also interesting to consider how EM fields may play a causal role in generating these signatures. Firstly, as has been recognized in many studies, conscious neuronal processing tends to be associated with re-entrant circuits, essentially closed loops of neuronal activity whereby neuronal outputs are fed back into input neurons. The function of these re-entrant circuits remains controversial, but they could be analogous to amplifier circuits in electronics that boost and phase-lock oscillations by feeding outputs back into inputs. However, in standard electronics, amplifier circuits respond linearly; whereas, as Dehaene and others have shown, consciousness demonstrates a non-linear all-or-nothing response in which increasing signal strength leads to a sudden transition from non-conscious to conscious perception. This is precisely the kind of behaviour—more like a phase transition—that would be expected for EM field-sensitive neuronal circuits that are acting as both transmitters and receivers of EM field information. If only a few neurons are firing synchronously then the EM fields generated by their firing will be too weak to influence firing. However, as more neurons are recruited into the re-entrant synchronously firing amplifier circuits then a threshold will be reached when the output EM field will be sufficiently strong to stimulate firing of multiple receiver neurons and thereby recruit more neurons into the amplifier network in a positive feedback loop. This EM field loop will rapidly amplify and expand the network of synchronous oscillations to create the kind of global ignition or neuronal avalanche that, by Dehaene and others, have described as a key signature of consciousness.

### The role of consciousness in learning and memory

It is well established that conscious awareness or attention appears to be a prerequisite to laying down long-term memories and for learning complex tasks (Baars and Gage 2010), but the mechanism remains obscure. Most theories account for this fact by simply incorporating a requirement for consciousness in laying down memories, without any physical justification for that rule. However, in the cemi field theory, a role for consciousness in memory and learning emerges as a natural consequence of the theory. For example, when learning a new motor skill, such as playing the piano, the small conscious pushes and pulls towards or away from neural firing (as anticipated by William James) provided by the brain’s endogenous EM field may be essential for delivering the fine motor control needed to hit the right notes at the right times. However, if the target neurones for EM augmentation are connected by Hebbian synapses then the influence of the brain’s EM field will tend to either increase [long-term potentiation (LTP)] or decreased [long-term depression (LTD)] neural connectivity: networks that deliver the skill will become hard-wired. After repeated augmentation by the brain’s EM field, conscious motor actions will become increasingly independent of EM field influences. The motor activity will be ‘learned’ in the sense that it will thereafter be capable of being performed without (conscious) EM field input. Indeed, because the networks will then be hard-wired into their optimal configuration, conscious EM field inputs will then tend to perturb the learned skill, exactly as we experience.

With only the recognition that EM fields influence neuron firing and the rule ‘neurons that fire together wire together’, a role for consciousness in memory thereby becomes inevitable. This proposed mechanism makes a clear prediction, that external EM fields will tend to interfere—positively or negatively—with memory and learning; which has been demonstrated in many TMS studies (Gagnon *et al.* 2011; Morgan *et al.* 2013).

### Objections to EM field theories of consciousness

I dealt with many of the most obvious objections to the cemi field theory in my earlier papers (McFadden 2002a,b), and Susan Pockett has reviewed most within an informative curated scholarpaedia page dedicated to EM field theories of consciousness (http://www.scholarpedia.org/article/Field_theories_of_consciousness). For example, it is often claimed that external EM fields incorrectly predict that external EM fields perturb our thoughts. As Pockett points out, this is easily refuted by the routine observation that they do not significantly influence EEG signals from the brain, presumably because their frequencies and strength do not couple with brain waves. The, by now, well-established neurophysiological (Pell *et al.* 2011) and cognitive (Guse *et al.* 2010; Rounis *et al.* 2010) effects of TMS do however provide strong evidence that, appropriately structured, external EM fields do indeed influence our thoughts. Nevertheless, whereas both evolution and development have, according to the cemi field theory, *tuned* the brain to both encode and decode EM field-based information to form coherent ideas, external EM fields from, say, TMS or MRI scanners, will, from the perspective of the brain, be incoherent signals, perhaps capable of disrupting, but not forming coherent thoughts.

Another common objection to EM field theories of consciousness is that ‘split brain’ patients with severed corpus callosum appear to possess two separated consciousnesses, despite, presumably, retaining a single intact brain EM field. However, although EM waves can indeed travel huge distance, their strength is subject to an inverse or cube law so that, as outlined above, EM field perturbations rapidly fall off with distance. For consciousness to be global, conscious information in EM fields thereby must to be amplified and transmitted by relays of in recurrent oscillations of synchronously firing neurons. It is these recurrent neuronal networks that are severed by the cutting of the corpus callosum in split brain patients, thereby preventing conscious EM field information in the right hemisphere from reaching speech centres in the left hemisphere (Fig. 3) and vice versa. Without the unification provided by networks of synchronously firing neurons, EM field information in each hemisphere will remain locked in each hemisphere.

Finally, there is the question why only some EM fields—those found inside brains—are conscious; whereas other EM fields, such as those generated by a toaster, are presumed to be non-conscious. Yet, just as people and toasters are made of matter but not all matter is alive, similarly, though consciousness may be made of EM fields, not all EM fields are conscious. Only a subset of systems made out of matter is alive; similarly only a subset of EM fields are likely to be conscious. The minimal characteristic of an EM field to qualify as conscious must surely be that it possesses sufficient complexity to encode complex computations together with causal power capable of transferring thoughts to another conscious being. Neither of these conditions is satisfied by the EM fields of a toaster or any other EM field, other than those inside brains. The cemi field theory thereby does not predict panpsychism for such objects.

### Testing the cemi field theory

Many of the predictions of the cemi field theory, such as that appropriately structured external EM fields will influence neural firing patterns and thoughts, have already been confirmed, as described above. The recent development of brain–computer interfaces (Mashat *et al.* 2017; Lazarou *et al.* 2018; Nuyujukian *et al.* 2018) that measure EEG signals and analyse those signals to generate limb motor outputs via TMS, effectively operates the same informational loop from neuron to neuron via the brain’s EM fields, as is proposed in the cemi field theory. Patients trained to use these devices experience EM field mediated motor control as their conscious actions. The cemi field theory merely proposes that the same information loop exists in all of us: we call it free will.

However, the cemi field theory also makes predictions that have yet to be tested, such as the possibility of inhibiting specific responses through specific external EM field perturbations. For example, it is well established that stimulus-provoked decision-making is accompanied by characteristic event related potentials (ERPs), such as the P300 (P3) wave that is associated with higher-level processing of incoming sensory information. The cemi field theory predicts that appropriately shaped artificial radio frequency or microwave EM fields that penetrate brain tissue should either reinforce of inhibit the motor response normally associated with the ERPs in a frequency and phase-dependent manner.

The cemi theory also has potentially transformative implications for the engineering of artificial consciousness. It accounts for why conventional computers, despite their undoubted computational skills, have not exhibited the slightest spark of consciousness, nor any signs of the general intelligence endowed by conscious minds. Many AI enthusiasts argue that artificial consciousness will emerge when computers eventually overtake the computational speed of the brain. The cemi theory predicts instead that no computer that computes solely through matter will ever be conscious, irrespective of its complexity, architecture or computational speed. The human brain is estimated to operate at about 1 exaFLOP capable of performing a billion billion calculations per second, about 20 times faster than the world’s fastest (in 2019) computer, the US Department of Energy’s Oak Ridge National Laboratory’s Summit, or OLCF-4 supercomputer. With computer speed continuing to follow Moore’s law, this prediction of the cemi field theory is likely to be tested within a decade or so.

However, although the cemi field theory insists that conventional computers will never be conscious, it does provide a route towards artificial consciousness through designing an EM field-sensitive computer. In fact, one may already have been constructed, albeit accidently. I previously described (McFadden 2002a) experiments performed by the School of Cognitive & Computing Sciences (COGS) group at the University of Sussex (Thompson *et al.* 1996; Davidson 1997), that group appears to have evolved an electronic circuit that computes through EM field interactions. The team used a silicon chip known as a field-programmable gate array (FPGA), comprised of an array of cells and software-configurable switches. Starting from a population of random configurations, the team selected for those better able to solve the toy task of distinguishing between two musical tones. After 5000 generations of this artificial selection, they succeeded in evolving a chip that could efficiently perform this task. However, when they examined its circuit diagram they discovered that some of its components which, if removed, impaired function, yet were not connected by wires to either inputs or outputs. Also, the performance of the chip was erratic and tended to work best at night. The solution to both these puzzles came from their realization that they had evolved the chip during experiments performed mostly at night when the researchers tended to listen to the radio. They concluded that their evolutionary process had not only optimized the wired connection of the chip, but also harnessed EMF coupling between the FPGA chip and the radio: they had evolved an algorithm in space, rather than time. I previously proposed (McFadden 2002a) that an analogous, though natural process, led to the evolution of consciousness in the human lineage. As far as I know, the approach of the COGS group has not been explored further; but, if the cemi field theory is correct, it provides a possible route towards building an artificial conscious mind.

### The physics of consciousness

Further insights into why we need EM fields to encode integrated conscious information can be gained directly from consideration of the physics of matter and energy. Matter is particulate whereas EM energy, such as light, is composed of waves. Nevertheless, the foundational experiments of quantum mechanics demonstrated that particles have wave-like properties and waves have associated particle properties. The information encoded in a particle is then also encoded in the wave associated with the particle. Physically unified integrated information could then potentially be encoded in matter, if their associated matter waves overlap. The wave-like properties of matter particles are however limited by their de Broglie wavelength, which is inversely proportional to their momentum (product of mass × velocity). So, an electron with rest mass energy of 0.511 MeV and kinetic energy of 1 eV will have an associated de Broglie wavelength of 1.23 nm. This is larger than the typical size of atoms, so electrons are delocalized in atomic orbitals of molecules such as benzene whose three pi electrons are delocalized across all six carbon atoms in the molecule. The electronic information encoded in the matter of electrons is thereby physically unified within molecules. However, the proton is about 1800 times more massive than an electron so its de Broglie wavelength and thereby its wave-like properties, when travelling at the same speed as the electron, is 1800 times smaller. The matter of protons is thereby localized entirely within the nucleus of each atom of a molecule such as benzene. Imagine writing either a 1 or 0 on each of three protons, or each of three pi electrons, in a single benzene molecule. To examine the three proton bits, it would be necessary to interrogate all three atoms within the molecule because each bit is locked within the de Broglie wavelength of each particle: their information is discrete and localized, not integrated. Yet all three electrons bits could be recovered by examining the pi electron configuration at any of the six atoms within the benzene ring: their information is delocalized and thereby integrated and unified across each of the atoms within the molecule. Essentially, each pi electron behaves like a wave within each benzene molecule: the information of all three electrons is *bound* within the molecule. Yet, this electron particle information molecule would not be available from an adjacent benzene molecule in, say, a crystal of benzene; unless the crystal is cooled to close to absolute zero so that the kinetic energy and thereby momentum of each particle is drastically reduced thereby lengthening its de Broglie wavelength and its wave-like properties, beyond individual molecules.

Since de Broglie wavelength is inversely proportionally to mass, molecules become more and more *particle-like* as they become more massive and detection of their wave nature, and thereby their ability to encode physically integrated information, becomes correspondingly difficult. Hence, although the wave properties of electrons was demonstrated in 1927, detecting the wave properties of a molecule consisting of up to 2000 atoms with de Broglie wavelengths of around 53 fm, five orders of magnitude smaller than the diameter of the molecule, was achieved only in 2019 in a stunning tour de force of interferometry (Fein *et al.* 2019).

**Figure 4:**
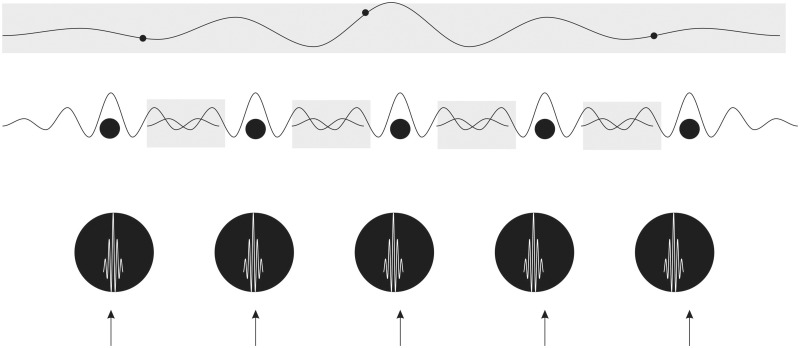
Particles, waves and integrated information. The wave-like behaviour of particles (black circles) is restricted to within their de Broglie wavelength which is illustrated (not to scale) for large mass particles, such as protons, atoms and molecules (bottom row), smaller mass particles, such as electrons (middle row), and massless particles, such as photons (top row, although, strictly, photons don’t really have a de Broglie wavelength or, if they do, it stretches to infinity). The areas of overlapping particle information, where information can be considered to be physically integrated, are indicated by grey shading.

However, under normal circumstances the information encoded within matter particles is integrated only within atoms and molecules, not between them (Fig. 4). This is what we mean by ‘matter’. So, information encoded in the matter particles of neurons, their ions, neurotransmitters or other biomolecules, is always discrete and localized within each molecule. This kind of information, although perfectly functional for temporal information processing, cannot be the substrate of physically integrated, unified and bound conscious information.

However, the situation is very different if, instead of the particles themselves, we consider the EM fields generated by charged particles, such as electrons. The EM field particle, the photon, has zero mass (photons do possess ‘relativistic mass’ but this is irrelevant to this argument.) so has no de Broglie wavelength. Instead, its wave potentially extends to infinity. The EM field of charged particles consist of virtual photons whose waves similarly extend to infinity, though decreasing in intensity according to an inverse square and cube laws (Fig. 4). Therefore, information encoded in charged particles of the brain, such as the ions involved in generating action potentials, is integrated, unified and bound within the overlapping EM fields generated by their motion. The brain’s EM field, rather than its matter, is thereby the only feasible physical substrate for conscious integrated information. Consciousness is what physically integrated information *feels like*, from the frame of the photons encoding that information.

## Discussion and Conclusions

Nagel famously asked us to imagine what it is like being a bat and insisted that we never can. The cemi field theory asks us to imagine what it would be like to be an EM field with inputs from oscillating electrons in neuronal membranes and outputs to oscillating electrons in neuronal membranes. Our tendency is to view this from a third person perspective looking down on the EM field and asking what its properties are. However, we must instead imagine moving to the frame of the brain’s EM field. This will be very different, analogous to how an EM field may be experienced as magnetic, from a stationary frame, but electric, from a moving frame, and *vice versa*. Einstein came up with special relativity through his *gedanken* experiment of considering what the universe would look like from the frame of a photon. Understanding awareness requires a similar shift to the frame of the EM field of the brain. We can, for example, consider what it would be like to be one photon in the cloud of photons (the cemi field) that travel from emitting to receiving electrons in neuronal membranes of the brain. From their frame, since they are massless particles that travel at the speed of light, they experience neither space nor time between emission and reception. Between these points, they may carry, say, up to 10 bits of information encoded in the photon’s energy, spin and direction (Tentrup *et al.* 2017). However, between emission and absorption, photons are more properly considered as delocalized waves that obey Maxwell’s laws. The cemi field is then the superposition of trillions of photon waves whose information is encoded in their ensuing pattern of constructive and destructive interference. That information is present at all points in the field, in the sense that the information encoded in a single photon emitted by an oscillating electron, say in the hypothalamus, can materialize—be absorbed by—any charged particle in its light cone, though with probability subject to the inverse square law and attenuation due to absorption events between emitter and receiver.

Nevertheless, within the brain, the light cones of all the trillions of emitted photon will almost entirely overlap. Any charged particle in the brain (or outside, but with rapidly diminishing probability) can potentially be the receiver of any of the trillions of emitted 10 bit packages of information available in the entire field. So, each point in the field represents the integrations of trillions of bits of physically encoded information instantaneously present at each point in the field. I previously proposed that ‘Nearly all qualia—the sound of C minor, the meaning of the number seven, the image of a triangle, the concept of a motor car, the feeling of anger, etc.—are similarly complex … conscious states [that] integrate parallel information streams to form a model that is both complex and physically unified within the cemi field.' That is, the qualia associated with hearing the musical note middle C is what an a EM field perturbation in the brain that correlates with the sensory input of middle C *feels like*, from the inside. IIT theory has also proposed that qualia are the experience of integrated information (Balduzzi and Tononi 2009); although Barrett argued that IIT cannot capture *intrinsic* (independent of an observer’s frame of reference) integrated information yet that could be captured by an EM field-based consciousness (Barrett 2014).

In this sense, the cemi theory incorporates Chalmers’ (Chalmers 1995) ‘double-aspect’ principle that information has both a physical, and a phenomenal or experiential aspect. At the particulate level, a molecule of the neurotransmitter glutamate encodes bond energies, angles, *etc*. but nothing extrinsic to itself. Awareness makes no sense for this kind matter-encoded information: what can glutamate be *aware of* except itself? Conversely, at the wave level, information encoded in physical fields is physically unified and can encode extrinsic information, as utilized in TV and radio signals. This EM field-based information will, according to the double-aspect principle, be a suitable substrate for experience. As proposed in my earlier paper (McFadden 2002a) ‘awareness will be a property of any system in which information is integrated into an information field that is complex enough to encode representations of real objects in the outside world (such as a face)’. Nevertheless, awareness is meaningless unless it can communicate so only fields that have access to a motor system, such as the cemi field, are candidates for any scientific notion of consciousness.

I previously proposed (McFadden 2013b), that complex information acquires its meaning, in the sense of binding of all of the varied aspects of a mental object, in the brain’s EM field. Here, I extend this idea to propose that meaning is an algorithm experienced, in its entirety from problem to its solution, as a single percept in the global workspace of brain’s EM field. This is where distributed information encoded in millions of physically separated neurons comes together. It is where Shakespeare’s words are turned into his poetry. It is also, where problems and solutions, such as how to untangle a rope from the wheels of a bicycle, are grasped in their entirety.

There are of course many unanswered questions, such as degree and extent of synchrony required to encode conscious thoughts, the influence of drugs or anaesthetics on the cemi field or whether cemi fields are causally active in animal brains. Yet the cemi theory provides a new paradigm in which consciousness is rooted in an entirely physical, measurable and artificially malleable physical structure and is amenable to experimental testing. The cemi field theory thereby delivers a kind of dualism, but it is a scientific dualism built on the distinction between matter and energy, rather than matter and spirit. Consciousness is what algorithms that exist simultaneously in the space of the brain’s EM field, *feel like*.

*Conflict of interest statement*. None declared.

## References

[niaa016-B1] Amassian VE , Cracco RQ, Maccabee PJ, et al Transcranial magnetic stimulation in study of the visual pathway. J Clin Neurophysiol 1998; 15:288–304. 9736464 10.1097/00004691-199807000-00002

[niaa016-B2] Anastassiou CA , Koch C. Ephaptic coupling to endogenous electric field activity: why bother? Curr Opin Neurobiol 2015; 31:95–103. 25265066 10.1016/j.conb.2014.09.002

[niaa016-B3] Anastassiou CA , Perin R, Markram H, et al Ephaptic coupling of cortical neurons. Nat Neurosci 2011; 14:217–23. 21240273 10.1038/nn.2727

[niaa016-B4] Anderson NH. Contributions to Information Integration Theory, Vol. 3. Developmental: Psychology Press, 2014.

[niaa016-B5] Arhem P , Johansson S. Spontaneous signalling in small central neurons: mechanisms and roles of spike-amplitude and spike-interval fluctuations. Int J Neural Syst 1996; 7:369–76. 8968826 10.1142/s0129065796000336

[niaa016-B6] Baars BJ. A Cognitive Theory of Consciousness. New York: Cambridge University Press, 1988.

[niaa016-B7] Baars BJ , Gage NM. Cognition, Brain, and Consciousness: Introduction to Cognitive Neuroscience. Burlington, MA, USA: Academic Press, 2010.

[niaa016-B8] Baars BJ. Global workspace theory of consciousness: toward a cognitive neuroscience of human experience. Progr Brain Res 2005; 150:45–53. 10.1016/S0079-6123(05)50004-9 16186014

[niaa016-B9] Balduzzi D , Tononi G. Qualia: the geometry of integrated information. PLoS Comput Biol 2009; 5:1–24. 10.1371/journal.pcbi.1000462 PMC2713405 19680424

[niaa016-B10] Banquet J-P. Spectral analysis of the EEG in meditation. Electroencephalogr Clin Neurophysiol 1973; 35:143–51. 4124606 10.1016/0013-4694(73)90170-3

[niaa016-B11] Barrett AB. An integration of integrated information theory with fundamental physics. Front Psychol 2014; 5:63. 24550877 10.3389/fpsyg.2014.00063 PMC3912322

[niaa016-B12] Beckers G , Hömberg V. Cerebral visual motion blindness: transitory akinetopsia induced by transcranial magnetic stimulation of human area V5. Proc R Soc Lond B Biol Sci 1992; 249: 173–8. 10.1098/rspb.1992.0100 1360678

[niaa016-B13] Carter RM , O’Doherty JP, Seymour B, et al Contingency awareness in human aversive conditioning involves the middle frontal gyrus. Neuroimage 2006; 29:1007–12. 16246595 10.1016/j.neuroimage.2005.09.011

[niaa016-B14] Chalmers DJ. Facing up to the problem of consciousness. J Conscious Stud 1995; 2:200–19.

[niaa016-B15] Davidson C. Creatures from primordial silicon - let Darwinism loose in an electronics lab and just watch what it creates. A lean, mean machine that nobody understands. New Sci 1997; 156:30–4.

[niaa016-B16] Dehaene S , Changeux J-P, Naccache L, et al Conscious, preconscious, and subliminal processing: a testable taxonomy. Trends Cogn Sci 2006; 10:204–11. 16603406 10.1016/j.tics.2006.03.007

[niaa016-B17] Dehaene S , Charles L, King J-R, et al Toward a computational theory of conscious processing. Curr Opin Neurobiol 2014; 25:76–84. 24709604 10.1016/j.conb.2013.12.005 PMC5635963

[niaa016-B18] Dehaene S , Cohen L. Cultural recycling of cortical maps. Neuron 2007; 56:384–98. 17964253 10.1016/j.neuron.2007.10.004

[niaa016-B19] Dehaene S. Consciousness and the Brain: Deciphering How the Brain Codes Our Thoughts. New York: Penguin, 2014.

[niaa016-B20] Dehaene S , Kerszberg M, Changeux J-P. A neuronal model of a global workspace in effortful cognitive tasks. Proc Natl Acad Sci USA 1998; 95:14529–34. 9826734 10.1073/pnas.95.24.14529 PMC24407

[niaa016-B21] Dehaene S , Naccache L. Towards a cognitive neuroscience of consciousness: basic evidence and a workspace framework. Cognition 2001; 79:1–37. 11164022 10.1016/s0010-0277(00)00123-2

[niaa016-B22] Doesburg SM , Kitajo K, Ward LM. Increased gamma-band synchrony precedes switching of conscious perceptual objects in binocular rivalry. Neuroreport 2005; 16:1139–42. 16012336 10.1097/00001756-200508010-00001

[niaa016-B23] Dor-Ziderman Y , Berkovich-Ohana A, Glicksohn J, et al Mindfulness-induced selflessness: a MEG neurophenomenological study. Front Hum Neurosci 2013; 7:582. 24068990 10.3389/fnhum.2013.00582 PMC3781350

[niaa016-B24] Dunbar R , Gamble C, Gowlett J. The Social Brain and its Distributed Mind. In: Social Brain, Distributed Mind. Oxford: Oxford University Press, p. 3–57, 2010.

[niaa016-B25] Edelman G , Tononi G. A Universe of Consciousness How Matter Becomes Imagination: How Matter Becomes Imagination. New York: Basic Books, 2008.

[niaa016-B26] Fein YY , Geyer P, Zwick P, et al Quantum superposition of molecules beyond 25 kDa. Nat Phys 2019; 1–4.

[niaa016-B27] Fingelkurts AA , Fingelkurts AA, Neves CF. Natural world physical, brain operational, and mind phenomenal space-time. Phys Life Rev 2001; 7:195–249. 10.1016/j.plrev.2010.04.001 20417160

[niaa016-B28] Fingelkurts AA , Fingelkurts AA. Brain-mind operational architectonics imaging: technical and methodological aspects. Open Neuroimag J 2008; 2:73–93. 19526071 10.2174/1874440000802010073 PMC2695620

[niaa016-B29] Frohlich F , McCormick DA. Endogenous electric fields may guide neocortical network activity. Neuron 2010; 67:129–43. 20624597 10.1016/j.neuron.2010.06.005 PMC3139922

[niaa016-B30] Fujisawa S , Matsuki N, Ikegaya Y. Chronometric readout from a memory trace: gamma-frequency field stimulation recruits timed recurrent activity in the rat CA3 network. J Physiol 2004; 561:123–31. 15375190 10.1113/jphysiol.2004.066639 PMC1665348

[niaa016-B31] Fuster J.M. Role of Prefrontal Cortex in delay tasks: Evidence from Reversible Lesion and Unit Recording in the Monkey. In: Levin HH, Eidenberg HM, Benton AL (eds) Frontal Lobe Function and Dysfunction. New York: Oxford University Press, 1991, pp. 59–71.

[niaa016-B32] Gagnon G , Schneider C, Grondin S, et al Enhancement of episodic memory in young and healthy adults: a paired-pulse TMS study on encoding and retrieval performance. Neurosci Lett 2011; 488:138–42. 21094215 10.1016/j.neulet.2010.11.016

[niaa016-B33] Genesereth MR , Keller AM, Duschka OM. *Infomaster: An Information Integration System. ACM SIGMOD Record.* ACM, 1997, 539–42.

[niaa016-B34] Guse B , Falkai P, Wobrock T. Cognitive effects of high-frequency repetitive transcranial magnetic stimulation: a systematic review. J Neural Transm 2010; 117:105–22. 19859782 10.1007/s00702-009-0333-7 PMC3085788

[niaa016-B35] Hagoort P , Indefrey P. The neurobiology of language beyond single words. Annu Rev Neurosci 2014; 37:347–62. 24905595 10.1146/annurev-neuro-071013-013847

[niaa016-B36] Hallett M. Transcranial magnetic stimulation and the human brain. Nature 2000; 406:147–50. 10910346 10.1038/35018000

[niaa016-B37] Han K-S , Guo C, Chen CH, et al Ephaptic coupling promotes synchronous firing of cerebellar Purkinje cells. Neuron 2018; 100:564–78. e3. 30293822 10.1016/j.neuron.2018.09.018 PMC7513896

[niaa016-B38] James W. Manuscript Lectures. Boston: Harvard University Press, 1988.

[niaa016-B39] John ER. A field theory of consciousness. Conscious Cogn 2001; 10:184–213. 11414714 10.1006/ccog.2001.0508

[niaa016-B40] John ER. The neurophysics of consciousness. Brain Res Brain Res Rev 2002; 39:1–28. 12086706 10.1016/s0165-0173(02)00142-x

[niaa016-B41] Kaufman AB , Kornilov SA, Bristol AS, Tan M, et al The neurobiological foundation of creative cognition. In: The Cambridge Handbook of Creativity. 2010, 216.

[niaa016-B42] Khrennikov A. Quantum-like model of processing of information in the brain based on classical electromagnetic field. Biosystems 2011; 105:250–62. 21683119 10.1016/j.biosystems.2011.05.014

[niaa016-B43] Kreiter AK , Singer W. Stimulus-dependent synchronization of neuronal responses in the visual cortex of the awake macaque monkey. J Neurosci 1996; 16:2381–96. 8601818 10.1523/JNEUROSCI.16-07-02381.1996 PMC6578521

[niaa016-B44] Landauer R. Information is physical. Phys. Today 1991; 44:23–9.

[niaa016-B45] Lazarou I , Nikolopoulos S, Petrantonakis PC, et al EEG-based brain–computer interfaces for communication and rehabilitation of people with motor impairment: a novel approach of the 21st century. Front Hum Neurosci 2018; 12:14. 29472849 10.3389/fnhum.2018.00014 PMC5810272

[niaa016-B46] Leon-Carrion J , Leon-Dominguez U, Pollonini L, et al Synchronization between the anterior and posterior cortex determines consciousness level in patients with traumatic brain injury (TBI). Brain Res 2012; 1476:22–30. 22534483 10.1016/j.brainres.2012.03.055

[niaa016-B47] Lieberman MD. What zombies can’t do: a social cognitive neuroscience approach to the irreducibility of reflective consciousness. 2012. Two Minds: Dual Processes and beyond. Oxford Scholarship Online, doi: 10.1093/acprof:oso/978199230167.003.0013.

[niaa016-B48] Lutz A , Lachaux J-P, Martinerie J, et al Guiding the study of brain dynamics by using first-person data: synchrony patterns correlate with ongoing conscious states during a simple visual task. Proc Natl Acad Sci USA 2002; 99:1586–91. 11805299 10.1073/pnas.032658199 PMC122234

[niaa016-B49] MacLennan BJ. Field computation in natural and artificial intelligence. Inf Sci 1999; 119:73–89.

[niaa016-B50] Maimon O , Rokach L. Data Mining and Knowledge Discovery Handbook. New York: Springer, 2005.

[niaa016-B51] Marcus G. Deep learning: a critical appraisal. *arXiv preprint arXiv : 180100631*, 2018.

[niaa016-B52] Mashat MEM , Li G, Zhang D. Human-to-human closed-loop control based on brain-to-brain interface and muscle-to-muscle interface. Sci Rep 2017; 7:11001. 28887545 10.1038/s41598-017-10957-z PMC5591235

[niaa016-B53] Mathewson KE , Lleras A, Beck DM, et al Pulsed out of awareness: EEG alpha oscillations represent a pulsed-inhibition of ongoing cortical processing. Front Psychol 2011; 2:99. 21779257 10.3389/fpsyg.2011.00099 PMC3132674

[niaa016-B54] McFadden J. Quantum Evolution. London: HarperCollins, 2000.

[niaa016-B55] McFadden J. Synchronous firing and its influence on the brain’s electromagnetic field: evidence for an electromagnetic theory of consciousness. J Conscious Stud 2002a; 9:23–50.

[niaa016-B56] McFadden J. The conscious electromagnetic information (Cemi) field theory: the hard problem made easy? J Conscious Stud 2002b; 9:45–60.

[niaa016-B57] McFadden J. The CEMI field theory closing the loop. J Conscious Stud2013a; 20:1–2.

[niaa016-B59] McFadden J. The CEMI field theory gestalt information and the meaning of meaning. J Conscious Stud 2013b; 20:152–82.

[niaa016-B60] Morgan HM , Jackson MC, van Koningsbruggen MG, et al Frontal and parietal theta burst TMS impairs working memory for visual-spatial conjunctions. Brain Stimul 2013; 6:122–9. 22483548 10.1016/j.brs.2012.03.001 PMC3605569

[niaa016-B61] Nuyujukian P , Sanabria JA, Saab J, et al Cortical control of a tablet computer by people with paralysis. PLoS One 2018; 13:e0204566. 30462658 10.1371/journal.pone.0204566 PMC6248919

[niaa016-B62] Pawłowski M , Paterek T, Kaszlikowski D, et al Information causality as a physical principle. Nature 2009; 461:1101–4. 19847260 10.1038/nature08400

[niaa016-B63] Pell GS , Roth Y, Zangen A. Modulation of cortical excitability induced by repetitive transcranial magnetic stimulation: influence of timing and geometrical parameters and underlying mechanisms. Progr Neurobiol 2011; 93:59–98. 10.1016/j.pneurobio.2010.10.003 21056619

[niaa016-B64] Pockett S. The Nature of Consciousness: A Hypothesis. Lincoln, NE: Writers Club Press, 2000.

[niaa016-B65] Pockett S. Difficulties with the electromagnetic field theory of consciousness. J Conscious Stud 2002; 9:51–6.

[niaa016-B66] Qiu C , Shivacharan RS, Zhang M, et al Can neural activity propagate by endogenous electrical field? J Neurosci 2015; 35:15800–11. 26631463 10.1523/JNEUROSCI.1045-15.2015 PMC4666910

[niaa016-B67] Quiroga RQ , Reddy L, Kreiman G, et al Invariant visual representation by single neurons in the human brain. Nature 2005; 435:1102. 15973409 10.1038/nature03687

[niaa016-B68] Rounis E , Maniscalco B, Rothwell JC, et al Theta-burst transcranial magnetic stimulation to the prefrontal cortex impairs metacognitive visual awareness. Cogn Neurosci 2010; 1:165–75. 24168333 10.1080/17588921003632529

[niaa016-B69] Ryle G. The Concept of Mind. London: Routledge, 2009.

[niaa016-B70] Schmitt RO , Dev P, Smith BH. Electrotonic processing of information by brain cells. Science 1976; 193:114–20. 180598 10.1126/science.180598

[niaa016-B71] Seth AK , Izhikevich E, Reeke GN, et al Theories and measures of consciousness: an extended framework. Proc Natl Acad Sci USA 2006; 103:10799–804. 16818879 10.1073/pnas.0604347103 PMC1487169

[niaa016-B72] Seth AK , McKinstry JL, Edelman GM, et al Visual binding through reentrant connectivity and dynamic synchronization in a brain-based device. Cerebral Cortex 2004; 14:1185–99. 15142952 10.1093/cercor/bhh079

[niaa016-B73] Shannon CE. A mathematical theory of communication. Bell Syst Technical J 1948; 27:379–423.

[niaa016-B74] Srinivasan R , Russell DP, Edelman GM, et al Increased synchronization of neuromagnetic responses during conscious perception. J Neurosci 1999; 19:5435–48. 10377353 10.1523/JNEUROSCI.19-13-05435.1999 PMC6782339

[niaa016-B75] Steinmann S , Leicht G, Ertl M, et al Conscious auditory perception related to long-range synchrony of gamma oscillations. Neuroimage 2014; 100:435–43. 24945670 10.1016/j.neuroimage.2014.06.012

[niaa016-B76] Sterzer P , Kleinschmidt A, Rees G. The neural bases of multistable perception. Trends Cogn Sci 2009; 13:310–8. 19540794 10.1016/j.tics.2009.04.006

[niaa016-B77] Tentrup TB , Hummel T, Wolterink TA, et al Transmitting more than 10 bit with a single photon. Optics Exp 2017; 25:2826–33. 10.1364/OE.25.002826 29518999

[niaa016-B78] Thompson A. Silicon evolution. In: Koza JR, et al (eds) Proceedings of Genetic Programming. Cambridge: MIT Press, 1996, 444–52.

[niaa016-B79] Tononi G , Edelman GM. Consciousness and complexity. Science 1998; 282:1846–51. 9836628 10.1126/science.282.5395.1846

[niaa016-B80] Tononi G , Srinivasan R, Russell DP, et al Investigating neural correlates of conscious perception by frequency-tagged neuromagnetic responses. Proc Natl Acad Sci USA 1998; 95:3198–203. 9501240 10.1073/pnas.95.6.3198 PMC19719

[niaa016-B81] Treisman A. Solutions to the binding problem: progress through controversy and convergence. Neuron 1999; 24:105–25. 10677031 10.1016/s0896-6273(00)80826-0

[niaa016-B82] Wilke M , Logothetis NK, Leopold DA. Local field potential reflects perceptual suppression in monkey visual cortex. Proc Natl Acad Sci USA 2006; 103:17507–12. 17088545 10.1073/pnas.0604673103 PMC1859959

[niaa016-B83] Wittgenstein L. Philosophical Investigations. Chichester, UK: John Wiley & Sons, 2009.

